# Evaluation of left ventricular ejection fraction using through-time radial GRAPPA

**DOI:** 10.1186/s12968-014-0079-8

**Published:** 2014-10-01

**Authors:** Gunhild Aandal, Vidya Nadig, Victoria Yeh, Prabhakar Rajiah, Trevor Jenkins, Abdus Sattar, Mark Griswold, Vikas Gulani, Robert C Gilkeson, Nicole Seiberlich

**Affiliations:** Radiology, Case Western Reserve University and University Hospitals Case Medical Center, Cleveland, OH USA; Haraldsplass Deaconess Hospital, Bergen, Norway; Cardiology, MetroHealth Medical Center at Case Western University, Cleveland, OH USA; Case Western Reserve University School of Medicine, Cleveland, OH USA; Division of Cardiovascular Medicine, Harrington Heart & Vascular Institute, University Hospitals Case Medical Center, Case Western Reserve University, Cleveland, OH USA; Epidemiology and Biostatistics, Case Western Reserve University, Cleveland, OH USA; Biomedical Engineering, Case Western Reserve University, Room 309 Wickenden Building 2071 Martin Luther King Jr. Drive, Cleveland, OH 44106-7207 USA

**Keywords:** Real-time imaging, Left ventricular ejection fraction, Cardiovascular magnetic resonance, Cardiac function

## Abstract

**Background:**

The determination of left ventricular ejection fraction using cardiovascular magnetic resonance (CMR) requires a steady cardiac rhythm for electrocardiogram (ECG) gating and multiple breathholds to minimize respiratory motion artifacts, which often leads to scan times of several minutes. The need for gating and breathholding can be eliminated by employing real-time CMR methods such as through-time radial GRAPPA. The aim of this study is to compare left ventricular cardiac functional parameters obtained using current gold-standard breathhold ECG-gated functional scans with non-gated free-breathing real-time imaging using radial GRAPPA, and to determine whether scan time or the occurrence of artifacts are reduced when using this real-time approach.

**Methods:**

63 patients were scanned on a 1.5T CMR scanner using both the standard cardiac functional examination with gating and breathholding and the real-time method. Total scan durations were noted. Through-time radial GRAPPA was employed to reconstruct images from the highly accelerated real-time data. The blood volume in the left ventricle was assessed to determine the end systolic volume (ESV), end diastolic volume (EDV), and ejection fraction (EF) for both methods, and images were rated for the presence of artifacts and quality of specific image features by two cardiac readers. Linear regression analysis, Bland-Altman plots and two-sided t-tests were performed to compare the quantitative parameters. A two-sample t-test was performed to compare the scan durations, and a two-sample test of proportion was used to analyze the presence of artifacts. For the reviewers´ ratings the Wilcoxon test for the equality of the scores’ distributions was employed.

**Results:**

The differences in EF, EDV, and ESV between the gold-standard and real-time methods were not statistically significant (p-values of 0.77, 0.82, and 0.97, respectively). Additionally, the scan time was significantly shorter for the real-time data collection (p<0.001) and fewer artifacts were reported in the real-time images (p<0.01). In the qualitative image analysis, reviewers marginally preferred the standard images although some features including cardiac motion were equivalently rated.

**Conclusion:**

Real-time functional CMR with through-time radial GRAPPA performed without ECG-gating under free-breathing can be considered as an alternative to gold-standard breathhold cine imaging for the evaluation of ejection fraction in patients.

## Background

Cardiovascular Magnetic Resonance (CMR) is considered to be the current gold-standard for the assessment of cardiac functional parameters, including left ventricular function [1,2]. While CMR has significant advantages over other imaging modalities, it can only be reliably used if the patient has a steady cardiac rhythm and the ability to perform the requisite breath-holds. These limitations restrict the patient populations that can be imaged with CMR, and can result in time-consuming and artifact-prone CMR examinations.

Real-time CMR has recently emerged as an alternative to standard CMR. In real-time CMR, imaging data are collected rapidly enough to effectively eliminate artifacts from cardiac or respiratory motion. Several real-time, non-breath-hold and non-ECG-gated imaging methods have been shown to be similar to the standard CMR methods in terms of image quality, and superior to echocardiography [3,4]. In order to achieve the high temporal resolution required (i.e. less than 50 ms per frame), many techniques have been investigated [5–15]. These techniques rely on data undersampling in conjunction with image reconstruction methods such as parallel imaging, compressed sensing, view-sharing, and retrospective navigation and/or registration. Real-time methods, while potentially effective for CMR without breath-holding or gating, are often hindered by challenges including low acceleration factors, the potential for temporal blurring, long reconstruction times, and the continued need for breath-holding to avoid motion artifacts. Additionally, many real-time methods have not been studied in large patient populations, and thus practical applicability remains to be determined. However, based on early studies, it has been shown that real-time cardiac imaging methods can provide significant new information for physicians, such as beat-to-beat or respiratory-dependent variations in motion or ventricular function [16].

Through-time radial GRAPPA is a real-time CMR technique that has been shown to provide robust image quality with temporal resolutions of less than 50 ms per frame [17]. This technique has been previously reported to allow high quality non-gated and free-breathing cardiac images in healthy volunteers by employing a radial data collection scheme in conjunction with a parallel imaging method based on GRAPPA [18]. Through-time radial GRAPPA offers several advantages over other real-time imaging techniques, including the ability to use high acceleration factors without relying on view-sharing or temporal regularization, which can lead to temporal blurring. Additionally, it has been shown that through-time radial GRAPPA reconstruction times can be less than 40 ms per frame using a Graphics Processing Unit (GPU) [19], which could allow for true real-time image collection and visualization at the CMR scanner. A previous study showed no significant differences between cardiac functional parameters when using standard breath-hold cine and highly accelerated through-time radial GRAPPA performed during a breath-hold in 20 patients [20].

While real-time free-breathing CMR using radial GRAPPA may prove useful for the assessment of functional parameters in patients who cannot currently be imaged with CMR, this technique cannot be employed without first validating the quantitative parameters obtained using this approach against gold-standard methods in patients who can be imaged with CMR. Thus, the primary purpose of this study is to compare quantitative left ventricular functional values determined using gold-standard breath-held and gated CMR images with those parameters obtained using free-breathing and ungated images reconstructed with through-time radial GRAPPA in a population of patients referred for CMR. The main hypothesis is that the functional parameters determined using free-breathing real-time imaging with through-time radial GRAPPA will be equivalent to those collected using gold-standard imaging. Additionally, the scan times needed to obtain images using the gold-standard method and the proposed real-time method were compared. Finally, the presence of artifacts and qualitative image ratings were compared in order to determine whether the use of the radial GRAPPA imaging technique leads to either fewer artifacts or a significant loss in visibility of image features.

## Methods

### Study population

This is a single-center, IRB-compliant prospective study. The study population included 63 consecutive patients undergoing routine CMR. At least 12 of the 63 patients were known to have experienced arrhythmias, at least 13 were known to have difficulty breath-holding, and at least two experienced both difficulties, although further information regarding the exact type of arrhythmia or poor breath-holding during the CMR scans was not available. Informed consent was obtained after the nature of the procedure had been fully explained. No patients above the age of 18 were excluded for reasons besides generally accepted contraindications for CMR in order to assure that the population was representative of patients undergoing CMR at our institution.

### CMR scanning

Subjects were scanned on a 1.5 T Avanto scanner (Siemens Medical Solutions, Erlangen, Germany) using a body and spine array coil combination (12 to 18 channels, depending on patient positioning). The gold-standard scan for the determination of functional parameters used a Cartesian bSSFP sequence with prospective ECG triggering and arrhythmia rejection with a parallel imaging acceleration factor of 2 and subsequent GRAPPA reconstruction. The left ventricle was covered in twelve to sixteen slices with separate breath-holds. In order to ensure that a fair comparison to the clinical standard was performed for each subject, parameters were optimized (as per the clinical protocol at our institution) for each subject according to their breath-holding capabilities and heart rate. The following ranges of parameters were employed: TR = 1.84-3.30 ms, TE = 0.92-1.65 ms, BW = 930-1502Hz/px, flip angle = 68-82°, read FoV = 230-450 mm, phase FoV = 228-366 mm, effective temporal resolution = 31-62 ms, in-plane resolution = 1.4-2.6 mm^2^, slice thickness = 6-8 mm, number of slices = 12-16, slice gap = 0-20%, cardiac phases = 13-33. The average temporal resolution (± standard deviation) was 40.3 ± 4.5 ms, and the average spatial resolutions in the phase and read directions were 1.81 ± 0.19 mm and 1.80 ± 0.19 mm.

The real-time, free-breathing scans were performed immediately following the standard scan with no ECG-gating or breath-holding. A radial bSSFP sequence was employed with the following sequence parameters: TR = 2.74 ms, TE = 1.37 ms, BW = 1115 Hz/px, flip angle = 70°, FoV = 300 mm^2^, temporal resolution = 43.8 ms, in-plane resolution = 2.3 mm^2^, acceleration factor of 8 with respect to Cartesian bSSFP (16 projections for 128^2^ matrix). The slice thicknesses, number of slices, and slice gaps were matched to the gold-standard scans used for each subject in order to provide the same coverage for both imaging methods. A total of 60 accelerated short-axis images were acquired for each slice in order to ensure capture of a complete cardiac cycle (approximately 2.5 seconds of imaging per slice).

The through-time radial GRAPPA reconstruction requires additional calibration data in the form of fully-sampled radial datasets. Thus, a total of either six (40 patients) or 26 (23 patients) fully-sampled calibration datasets for each slice were also acquired directly following the acquisition of the real-time data. These calibration scans had sequence parameters as described above, with the exception of the number of projections, which was set to 144 to form a fully-sampled radial dataset. The slice thicknesses, number of slices, and slice gaps were matched to the real-time data and gold-standard data. This calibration phase lasted an additional 2.4 seconds (six calibration frames) or 10.3 seconds (26 calibration frames) per slice. All calibration and accelerated radial data were acquired without cardiac gating during free-breathing. The start and end times of the standard breath-hold scans and the real-time free-breathing scans (calibration times included) were noted for each subject.

### Image reconstruction

The gold-standard cardiac images were collected from the scanner in DICOM format, with no further post-processing. The real-time radial data were exported to an off-line computer and reconstructed into images using through-time radial GRAPPA (Matlab R2011b, The Mathworks, Natick, MA). The radial GRAPPA reconstruction kernel size was set to 2×3 (projection × read) for all reconstructions, as described in [17]. Similarly, segment sizes of 8×4 (read × projection) were used for the reconstruction when 26 calibration frames were available, and this segment size was increased to 16×8 when only six calibration frames were collected. The segment size for the longer calibration scan was determined based on results from the original through-time radial GRAPPA paper [17]. The shorter calibration scan was selected to be 6 frames to equalize the calibration and accelerated imaging times. The reconstruction parameters for the shorter calibration time were chosen to keep the number of equations for the GRAPPA weight determination approximately equal for both the long and the short calibration schemes.

**Figure 1:**
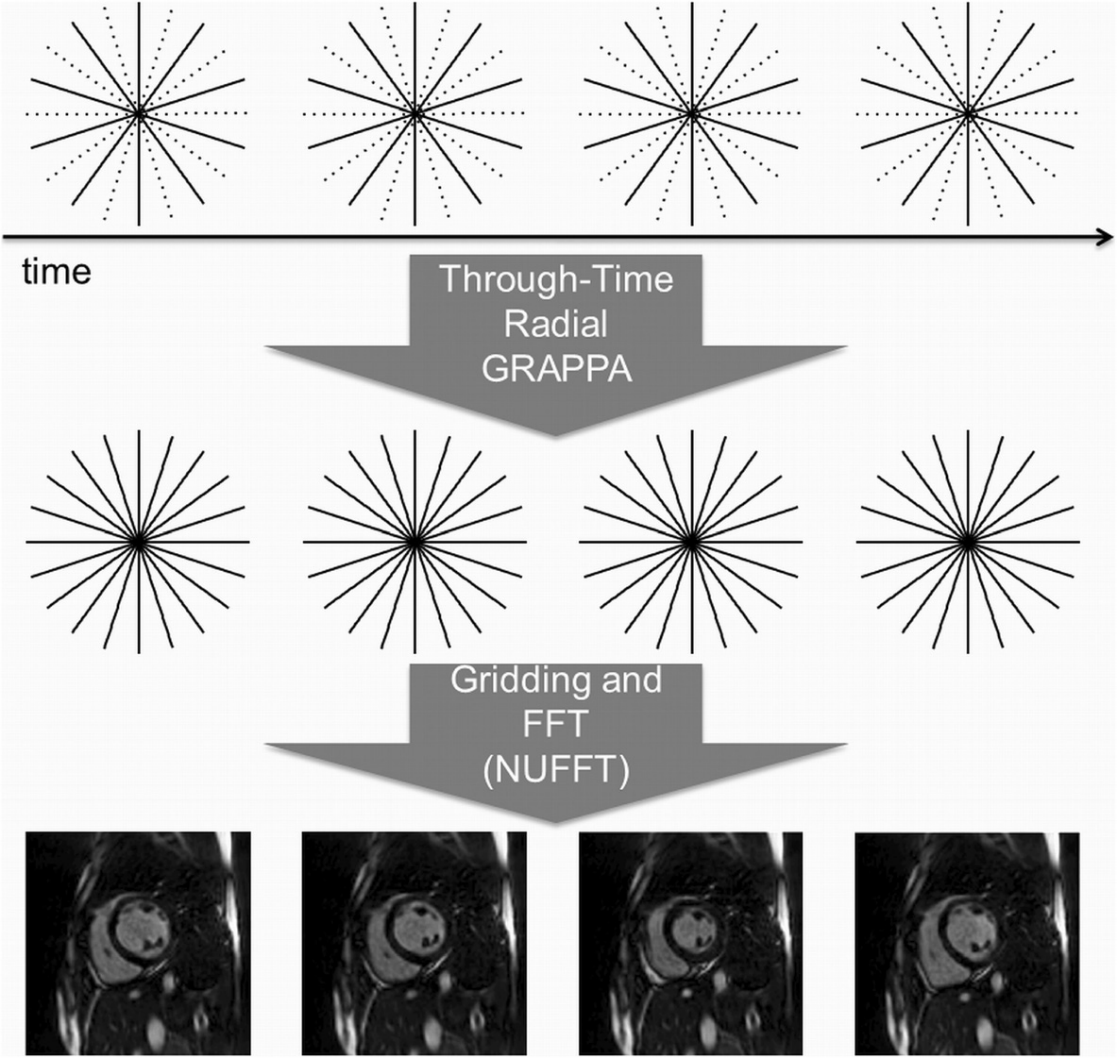
**A schematic of the reconstruction pipeline for real-time imaging with radial GRAPPA.** Top: Highly accelerated radial k-space data are acquired for a high temporal resolution. Middle: Missing radial lines of data are reconstructed using through-time radial GRAPPA, where the weights are determined using the calibration scan. Bottom: Images are generated from the reconstructed fully-sampled radial data using NUFFT.

A schematic overview of the through-time radial GRAPPA reconstruction is shown in Figure 1. The GRAPPA weights generated from the fully-sampled data are applied to the undersampled radial data (Figure 1, top) to reconstruct fully-sampled radial data (Figure 1, middle), then transformed to the image domain (Figure 1, bottom) using the NUFFT [21]. The reconstruction required approximately three minutes per slice in the off-line implementation.

### Quantitative parameter determination

The blood volume in the left ventricle for each of the datasets was assessed by a single physician to determine the end-diastolic volume (EDV), end-systolic volume (ESV) and ejection fraction (EF) for both imaging techniques. For the standard gated CMR images, only one composite heartbeat is available for analysis. The end-diastolic frame for the gold-standard images was determined as the frame with the largest blood pool volume, and the end-systolic frame was determined as the frame with the smallest blood pool volume, automatically using Argus Ventricular Function software (Siemens Medical Solutions). For the real-time images, sixty cardiac images spanning several cardiac cycles were collected for each slice. The first full cardiac cycle acquired for each slice was chosen and EDV and ESV were determined for each individual slice using Argus as described above. No control for breath-hold state or image registration was performed.

### Qualitative image review

After data reconstruction, the anonymized images were presented to two board-certified cardiac imagers with seven and one year experience respectively for independent review. The images were rated on a scale of excellent (4), good (3), poor (2), no visibility (1). The readers were asked to rate the following features: endocardial border definition, mitral valve and papillary muscle visualization, visualization of myocardium, blood pool contrast, and cardiac motion. Images were also reviewed for the presence of artifacts (including artifacts due to respiration or misgating, as well as parallel imaging reconstruction and radial streak artifacts). Artifacts were graded on a 5 point scale: no artifact (1); minimal artifact not affecting volumetric analysis (2), mild artifact affecting volumetric analysis (3), moderate artifact affecting volumetric analysis (4); extensive artifact affecting volumetric analysis (5).

### Statistical analysis

Statistical analysis was performed using commercially available software (Excel 12.3.6, Stata 11.2). Linear regression analyses were performed and Bland-Altman plots [22] were generated to evaluate the agreement between the two methods in the estimation of EDV, ESV and EF. Two-sided t-tests with unequal variance assumption were used in testing the equality of means of EDV, ESV and EF measures obtained from the two methods. A two-sample t-test was performed to compare the scan durations between the gold-standard scans and the real-time scans. Average scores for each metric of reviewer ratings were computed and the distributions were compared using the Wilcoxon test for the equality of the scores’ distributions. Finally, a two-sample test of proportion was used to analyze the presence and absence of artifacts.

## Results

**Figure 2:**
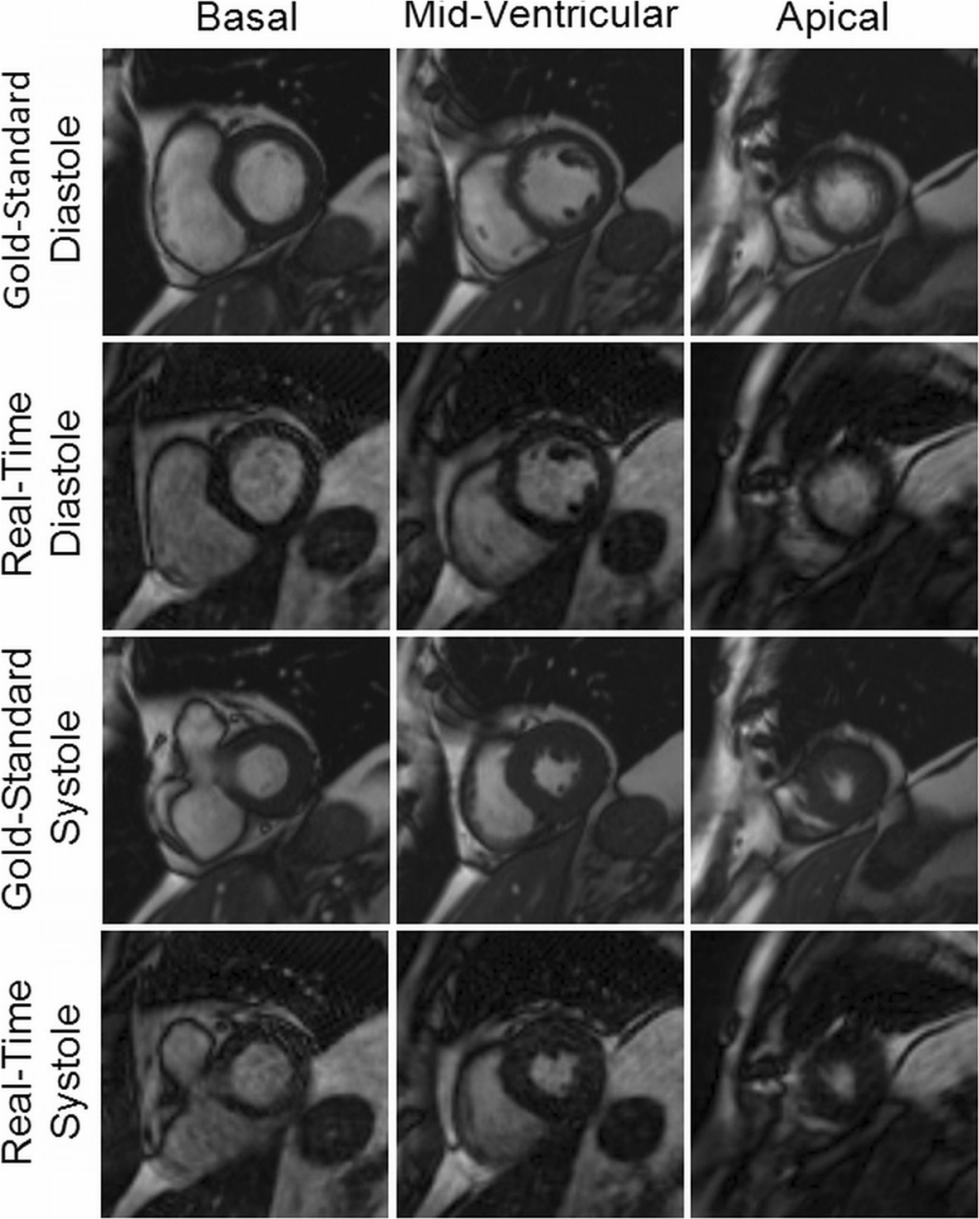
**Example images from a patient acquired using both the gold-standard breath-hold and ECG-gated cine (first and third rows) and the real-time imaging technique (second and fourth rows) for three different slice locations in both end-diastole and end-systole.** Ejection fraction values of 68.8% and 66.7% were determined from the standard and real-time images, respectively. Both sets of images were rated to be artifact-free, and the gold-standard images were rated as “excellent” in all categories by both reviewers. The real-time images were rated as “excellent” in every category by Reviewer 1, and “excellent” in three categories and “good” in the remaining three by Reviewer 2.

**Figure 3:**
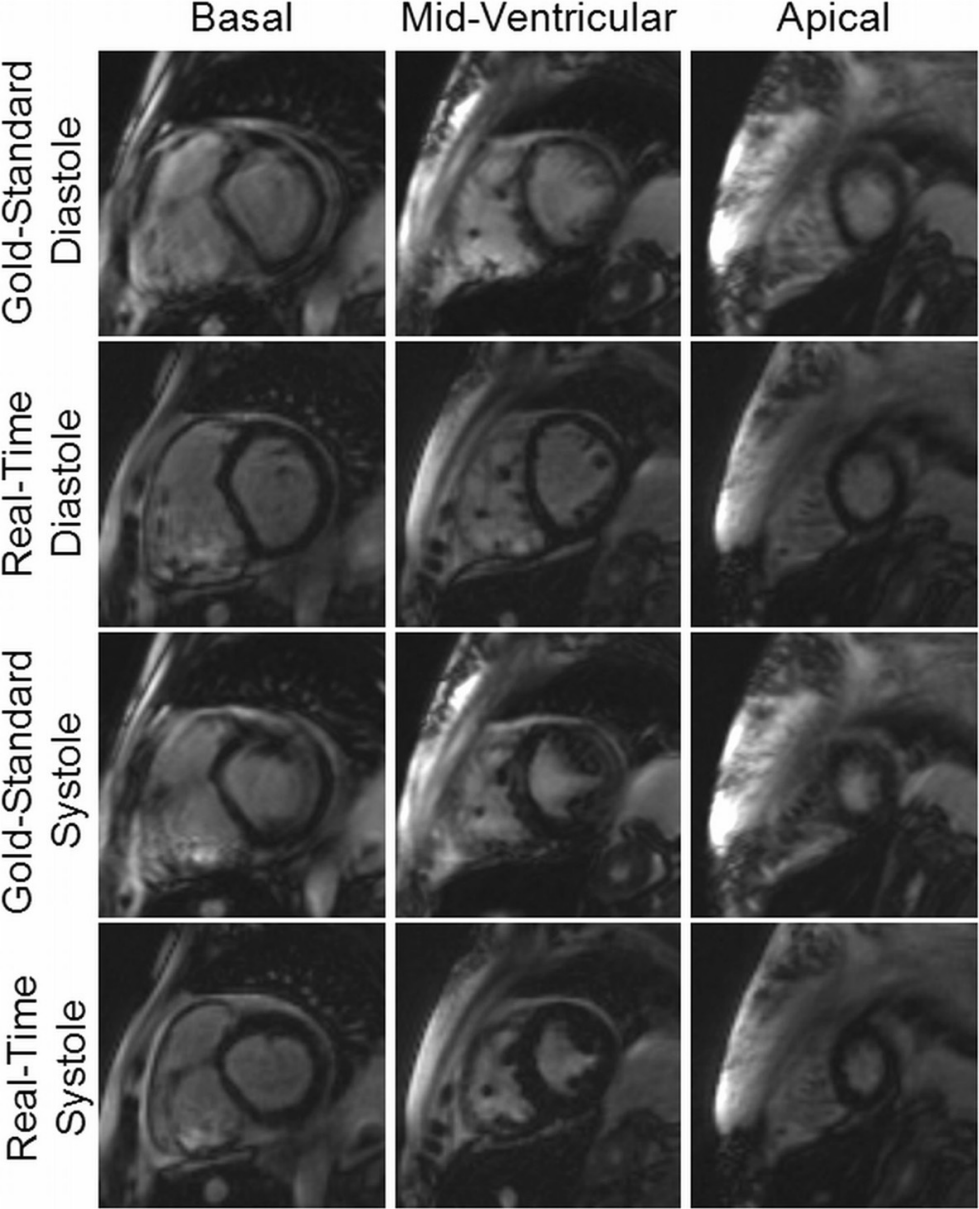
**Example images from a subject with arrhythmia and prolonged breath-hold acquired using both the gold-standard breath-hold and ECG-gated cine (first and third rows) and the real-time non-gated and free-breathing imaging technique (second and fourth rows) for three different slice locations in both end-diastole and end-systole; note the significant artifacts in the gold-standard images (as noted by the reviewers) and the comparatively sharp endocardial border definition in the real-time images.** The EF was found to be 33.9% and 35.2% for the standard and real-time measurements, respectively. The real-time images were rated better in every category than the gold-standard images by the reviewers; all but one of the ratings for the real-time images were “excellent”, whereas more than half of the gold-standard ratings were “good” and the rest “excellent”.

Example images collected using both the gold-standard breath-hold gated scan as well as the real-time, free-breathing and ungated images with radial GRAPPA are shown in Figures 2 and 3. For each patient, multiple slice positions in end-systole and end-diastole are shown in order to demonstrate the relative image quality.

For the entire subject population, the average and standard deviations (mean ± SD) for the EF, EDV, and ESV as determined using the standard functional imaging method were found to be 48.0% ± 15.6%, 140.7 mL ± 68.1 mL, and 79.2 mL ± 60.0 mL, respectively. The average and standard deviations for EF, EDV, and ESV as determined using radial GRAPPA were similar, measuring 48.8% ± 15.0%, 143.3 mL ± 67.9 mL, and 79.0 mL ± 59.5 mL, respectively. The average and standard deviations for the differences in EF, EDV, and ESV between the two methods were also calculated and found to be −0.9% ± 2.3%, −2.6 mL ± 13.5 mL, and 0.3 mL ± 8.8 mL, respectively. These values are summarized in Table 1. In order to determine whether the number of calibration frames used for the radial GRAPPA reconstruction affected these averages and standard deviations, these values were calculated separately for these two groups and also reported in Table 1. The largest absolute difference in EF between the two methods in a single subject was 7.2%, and the variance for the differences in the two EF measurements was 5.1%.

**Table 1:** **Summary of Quantitative Assessment**

	**Gold-standard**	**Real-time**
**Entire subject population (63/63)**		
EDV	140.7 mL ± 68.1 mL	143.3 mL ± 67.9 mL
ESV	79.2 mL ± 60.0 mL	79.0 mL ± 59.5 mL
EF	48.0% ± 15.6%	48.8% ± 15.0%
**Radial GRAPPA—26 cal frames (23/63)**		
EDV	131.9 mL ± 72.5 mL	131.5 mL ± 68.7 mL
ESV	77.3 mL ± 62.3 mL	74.4 mL ± 58.9 mL
EF	46.6% ± 17.1%	48.3% ± 16.6%
**Radial GRAPPA—6 cal frames (40/63)**		
EDV	145.8 mL ± 65.8 mL	150.1 mL ± 67.4 mL
ESV	80.4 mL ± 59.4 mL	81.6 mL ± 60.4 mL
EF	48.7% ± 14.7%	49.1% ± 14.2%
**Ejection fraction > 50% (33/63)**	59.3% ± 8.5%	59.6% ± 8.2%
**40% < Ejection fraction < 50% (14/63)**	44.5% ± 3.3%	46.0% ± 2.7%
**30% < Ejection fraction < 40% (9/63)**	34.9% ± 2.6%	36.8% ± 3.5%
**Ejection fraction < 30% (7/63)**	18.2% ± 6.9%	19.0% ± 5.4%

Summary of average and standard deviations of ESV, EDV, and EF values for the study population as a whole, divided into subcategories with different numbers of calibration frames for the radial GRAPPA reconstruction, and sorted into EF ranges.

A total of 33 of the 63 subjects had EF values above 50%, 14 had EF values between 40-50%, 9 had EF values between 30-40%, and 7 had EF values of less than 30%. The average and standard deviations for the ESV, EDV, and EF values broken down into these EF ranges for both the gold-standard and real-time radial GRAPPA scans are also shown in Table 1 so that the two methods can be compared in patients with different levels of cardiac function. Of the 63 patients, two patients crossed the 35% EF threshold commonly used to determine EF dysfunction. For the first of these patients, the breath-hold EF value was 34.0% and the real-time EF was 35.4%, and for the second patient, the breath-hold EF was 33.9% and the real-time EF was 35.2%. Please note that the images for this second patient are shown in Figure 3.

**Figure 4:**
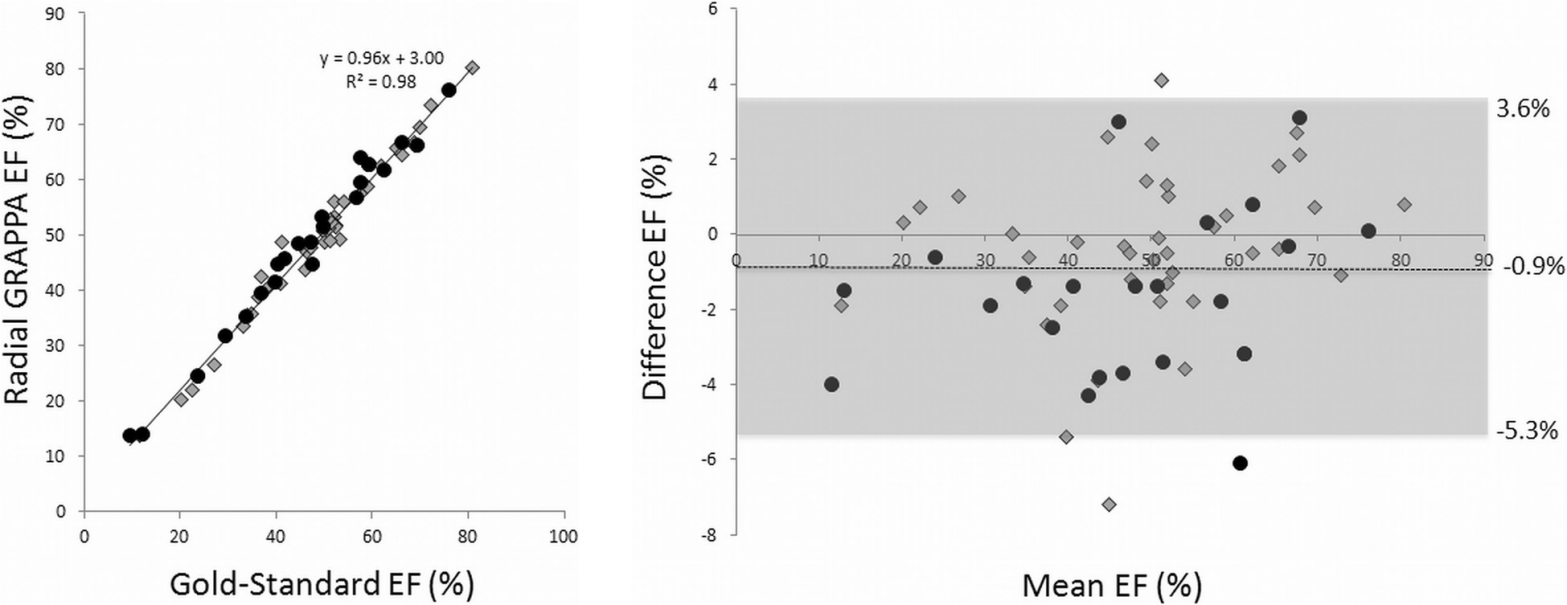
**Linear regression (left) and Bland-Altman plot (right) for the ejection fraction values.** Values from all 63 subjects are included in both plots. Gray diamonds denote subjects for which only 6 calibration frames were used when performing the radial GRAPPA reconstruction, and black circles denote subjects for which 26 calibration frames were used. In the Bland-Altman plot, the mean of the two measurements is plotted on the x-axis, and the difference (gold-standard – radial GRAPPA) on the y-axis. The mean difference and 95% limits of agreement are noted on the plot.

**Figure 5:**
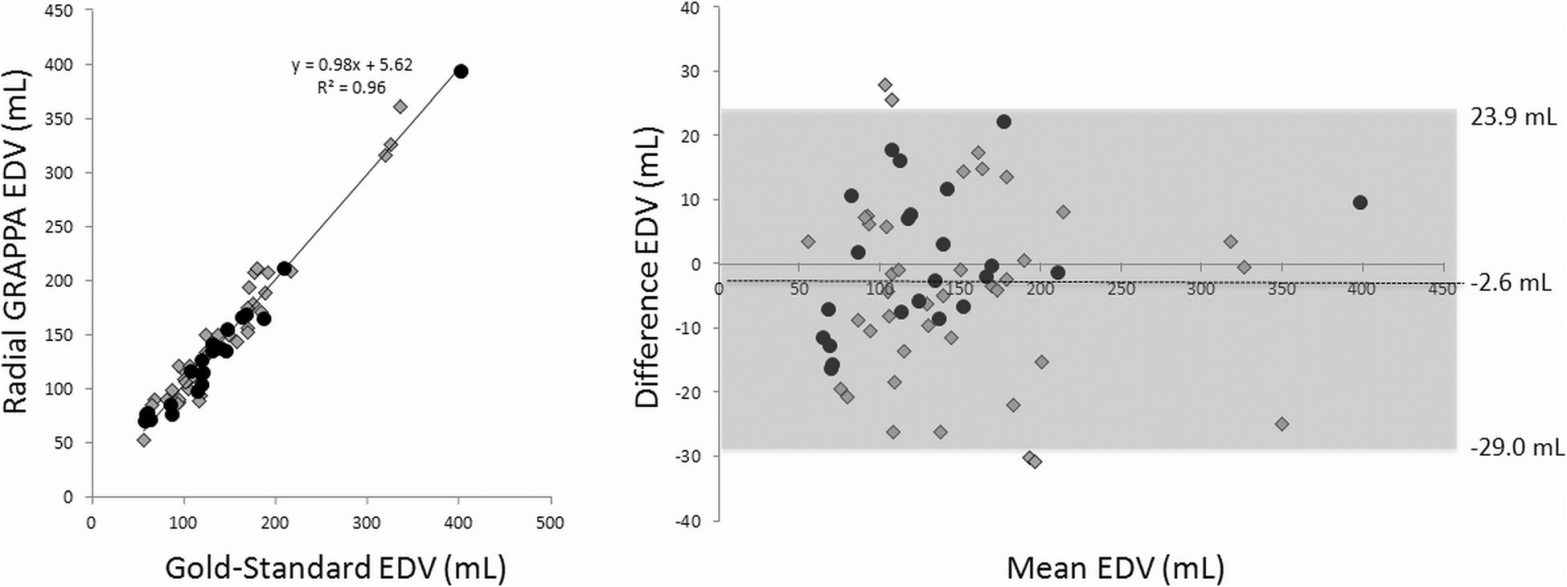
**Linear regression and Bland-Altman plot for the EDV values.** Values from all 63 subjects are included in both plots. Gray diamonds denote subjects for which only 6 calibration frames were used when performing the radial GRAPPA reconstruction, and black circles denote subjects for which 26 calibration frames were used. In the Bland-Altman plot, the mean of the two measurements is plotted on the x-axis, and the difference (gold-standard – radial GRAPPA) on the y-axis. The mean difference and 95% limits of agreement are noted on the plot

**Figure 6:**
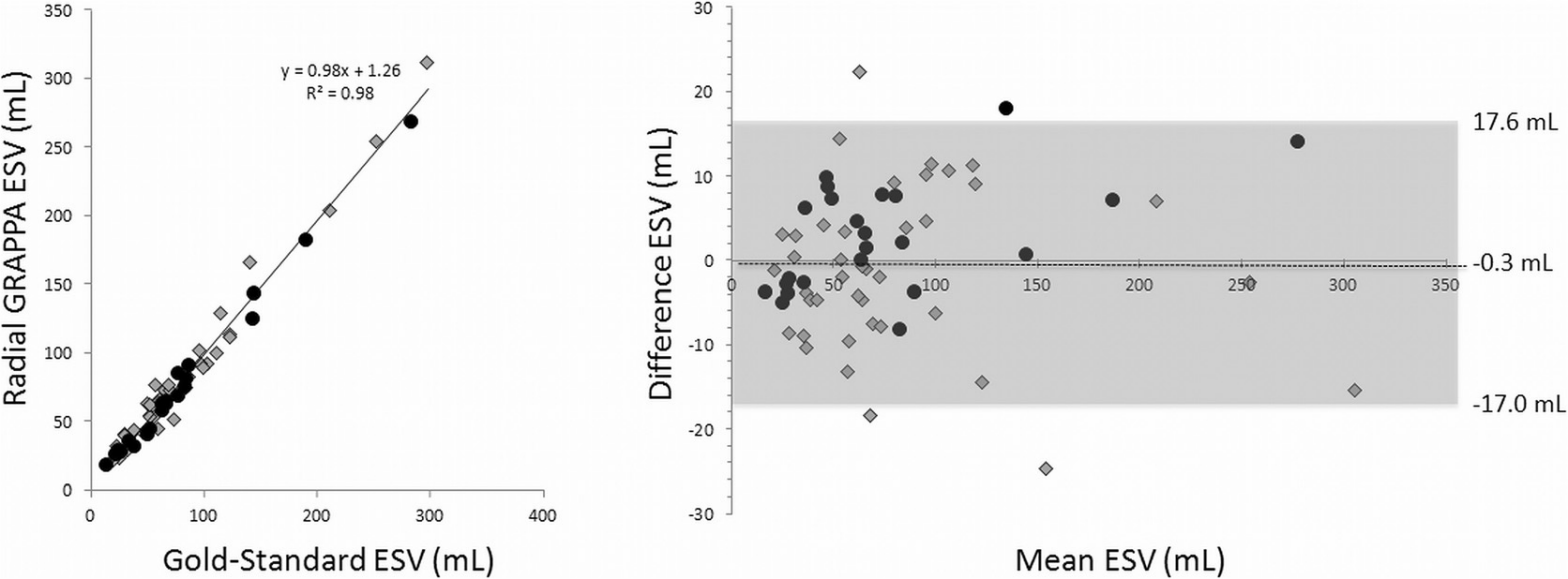
**Linear regression and Bland-Altman plot for the ESV values.** Values from all 63 subjects are included in both plots. Gray diamonds denote subjects for which only 6 calibration frames were used when performing the radial GRAPPA reconstruction, and black circles denote subjects for which 26 calibration frames were used. In the Bland-Altman plot, the mean of the two measurements is plotted on the x-axis, and the difference (gold-standard – radial GRAPPA) on the y-axis. The mean difference and 95% limits of agreement are noted on the plot

The linear regression and Bland-Altman plot for the ejection fraction measurements are shown in Figure 4. For each plot, the datapoints generated using radial GRAPPA with six calibration frames are shown as gray diamonds, and those generated using radial GRAPPA with 26 calibration frames are shown as black circles. All values reported here were determined using all 63 subjects, and similar values broken down by number of calibration frames are given in Table 2. For EF, the overall R^2^ value for the linear regression was 0.98. For the Bland-Altman plot, the 95% limits of agreement (−5.3%, 3.6%) contained 95.2% (60/63) of the difference scores. The mean difference (bias) of the measurements between the gold-standard and real-time methods was −0.9%, and the maximum and minimum differences were 4.2% and −7.2% respectively. The linear regression and Bland-Altman plots for EDV and ESV are shown in Figures 5 and 6. The R^2^ values were 0.96 and 0.98 for the EDV and ESV, respectively. The Bland-Altman analysis of the EDV data showed that the 95% limits of agreement (−29.0 mL, 23.9 mL) contains 93.6% (59/63) of the difference scores, and the mean difference of the measurements was −2.6 mL. Similarly, the 95% limits of agreement of ESV measurements (−17.0 mL, 17.6 mL) contains 93.6% (59/63) of the difference scores, and the mean difference was 0.3 mL. For all three quantitative parameters, namely EF, EDV and ESV, the mean differences are small (approximately 1%) and limits of agreement are narrow, indicating that the two methods are systematically producing similar results. The hypothesis tests of equal mean show that the means of EF, EDV, and ESV obtained from the two methods are equivalent with p-values 0.77, 0.82, and 0.97, respectively.

**Table 2:** **Summary of Results of Statistical Analysis of Quantitative Parameters**

	**Ejection fraction**	**End diastolic volume**	**End systolic volume**
**For all subjects (63/63)**			
R^2^	0.98	0.96	0.98
Mean difference	−0.9%	−2.6 mL	0.3 mL
Lower 95% limit of agreement	−5.3%	−29.0 mL	−17.0 mL
Upper 95% limit of agreement	3.6%	23.9 mL	17.6 mL
p-value	0.77	0.82	0.97
**Radial GRAPPA with 26 calibration frames (23/63)**			
R^2^	0.98	0.98	0.99
Mean difference	−1.7%	0.4 mL	2.9 mL
Lower 95% limit of agreement	−6.1%	−21.0 mL	−10.1 mL
Upper 95% limit of agreement	2.7%	21.7 mL	15.9 mL
p-value	0.73	0.99	0.87
**Radial GRAPPA with 6 calibration frames (40/63)**			
R^2^	0.98	0.95	0.97
Mean difference	−0.4%	−4.3 mL	−1.2 mL
Lower 95% limit of agreement	−4.7%	−33.1 mL	−20.1 mL
Upper 95% limit of agreement	3.9%	24.5 mL	17.7 mL
p-value	0.90	0.78	0.93

A summary of the R^2^ values from the regression analysis, the mean differences and upper and lower 95% limits of agreement from the Bland-Altman plots, and the p-values from the hypothesis tests of equal mean for the EF, ESV, and EDVs calculated using the two different imaging methods. The top third of the table shows values derived from all subjects, while the central and bottom thirds show the values when using only subsets of the subjects with the same number of calibration frames for the radial GRAPPA reconstructions.

The start and end times for both the gold-standard and radial GRAPPA scans were recorded for all subjects. The mean and standard deviation of the scan duration for the gold-standard functional imaging was 5.8 ± 1.9 minutes. For the real-time radial GRAPPA scans using 26 calibration frames, the mean and standard deviation of the scan duration was 4.6 ± 0.8 minutes, and for the radial GRAPPA scans using six calibration frames, the mean and standard deviation of the scan duration was 2.8 ± 0.4 minutes. Based on the two-sample t-test, the scan time was shortened by a statistically significant amount when using the real-time method (p < 0.001) either with 26 or six calibration frames.

Out of the 63 subjects, reviewer one indicated that 30 of the gold-standard (score 1.8 ± 0.9) and none of the radial GRAPPA scans (1 ± 0) exhibited artifacts and reviewer two indicated that 13 gold-standard (1.3 ± 0.6) and two real-time scans (with 26 calibration frames) (1.0 ± 0.1) contained such artifacts. The two-sample test of proportion showed that this is a statistically significant difference (p < 0.01), indicating that the use of real-time imaging in the form of radial GRAPPA reduces the number of image exhibiting artifacts.

**Figure 7:**
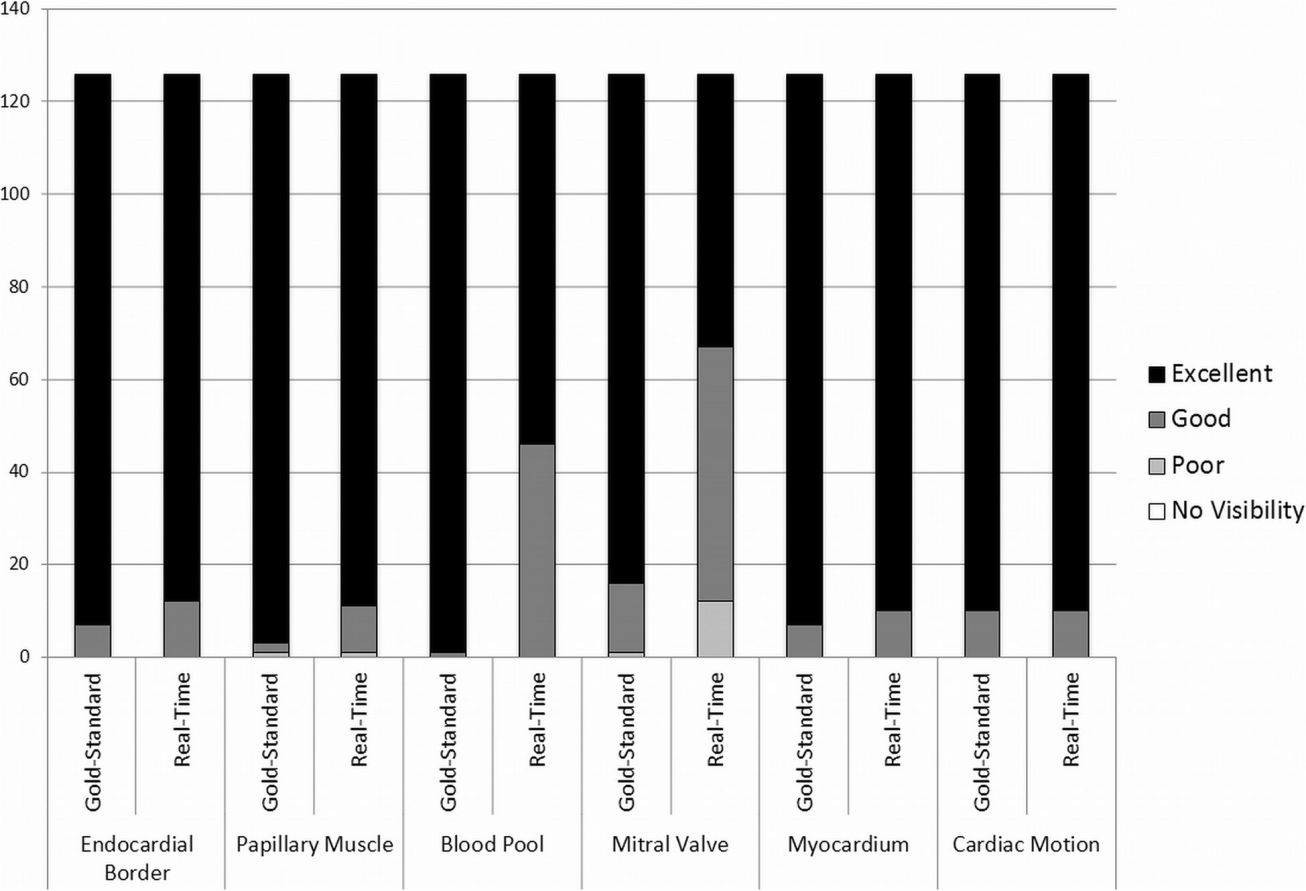
**A summary of the reviewer ratings for each of the six image feature categories for all subjects for both the gold-standard and the radial GRAPPA images.**

Results of the qualitative image feature ratings and statistical analysis are summarized in both Table 3 and Figure 7. As indicated in Table 3, the overall averages in each category were above 3, indicating that the feature visibility for both types of imaging methods was good to excellent. Figure 7 shows that the ratings were almost exclusively chosen to be 3 or 4 (“good” or “excellent”) although there were some exceptions. For instance, both reviewers found the mitral valves to be more poorly visualized in the radial GRAPPA images in comparison to the standard images. One reviewer indicated that papillary muscle visualization was “poor” for both imaging techniques in one subject, although the rest of the ratings were in the “good” and “excellent” categories. No other features besides these two were rated as “poor” (or 2) for either type of scan, and no features for either type of image were rated as “not visible” (or 1) by either reader.

**Table 3:** **Summary of Reviewer Ratings**

	**Average gold-standard**	**Average real-time**	**Wilcoxon p-value**
**For all subjects (63/63)**			
Endocardial border	3.9	3.9	0.23
Papillary muscle	4.0	3.9	0.03*
Blood pool	4.0	3.6	<0.001*
Mitral valve	3.9	3.4	<0.001*
Myocardium	3.9	3.9	0.45
Cardiac motion	3.9	3.9	1.00
**Radial GRAPPA with 26 calibration frames (23/63)**			
Endocardial border	3.9	3.9	0.73
Papillary muscle	4.0	4.0	0.71
Blood pool	4.0	3.7	<0.01*
Mitral valve	3.8	3.2	<0.001*
Myocardium	3.9	4.0	0.17
Cardiac motion	3.9	4.0	0.14
**Radial GRAPPA with 6 calibration frames (40/63)**			
Endocardial border	4.0	3.9	0.05
Papillary muscle	4.0	3.9	0.02*
Blood pool	4.0	3.6	<0.001*
Mitral valve	3.9	3.5	<0.001*
Myocardium	4.0	3.9	0.07
Cardiac motion	4.0	3.9	0.23

Average results and p-values of the reviewer ratings for image features. The subject population was examined as a whole, and statistics were also calculated for the two subsets of images with different numbers of calibration frames for the radial GRAPPA reconstruction. Stars denote statistically significant differences.

Statistical analyses using the Wilcoxon test were performed using all 63 subjects together, and also on each subgroup with different numbers of calibration frames for the radial GRAPPA reconstruction. The Wilcoxon tests showed that the image quality scores for the myocardium (p = 0.45 overall, p = 0.07 with 6 calibration frames, p = 0.17 with 26 calibration frames) and cardiac motion (p = 1.00 overall, p = 0.23 with 6 calibration frames, p = 0.14 with 26 calibration frames) follow the same distribution; this indicates that neither set of images was preferred for these features. The reviewers strongly preferred the standard images for blood pool contrast (p < 0.001 overall and with 6 calibration frames, and p < 0.01 with 26 calibration frames) and mitral valve visualization (p < 0.001 overall and for both subgroups). For the visualization of the endocardial border, there was no statistically significant difference between the gold-standard scans and the radial GRAPPA reconstructions when 26 calibration frames were used (p = 0.73), However, when only six calibration frames were used for the radial GRAPPA, the p-value drops substantially (p = 0.05). Similar results are found for the visualization of the papillary muscles, where the image ratings show no statistically significant differences when 26 calibration frames are (p = 0.71), but significant differences when only six frames are used (p = 0.02).

## Discussion

This study demonstrates that through-time radial GRAPPA used for real-time, free-breathing CMR imaging produces equivalent results for volumetric analysis of the left ventricle when compared to the current gold-standard CMR imaging technique. The high R^2^ values in the linear regressions, small mean differences and narrow 95% limits of agreement in the Bland-Altman plots, and large p-values for EF, EDV, and ESV indicate that the radial GRAPPA images can be used to obtain similar volumetric information as the standard scans. The two types of scans are equivalent regardless of the number of calibration frames used for the radial GRAPPA reconstruction; reconstructions with 26 and six frames have high R^2^ and p-values. Prior studies [23–27] have indicated that ejection fraction measurements with a variance of 10% can be considered equivalent, and the data presented here have a smaller variance of only 5.1%. Additionally, a comparison of average and standard deviations of the ESV, EDV, and EF values measured using the gold-standard methods and the real-time approach for subjects sorted into ranges of EF > 50%, 40% < EF < 50%, 30% < EF < 40%, and EF < 30% shows that these values are similar for all ranges. These results indicate that the real-time approach offers similar volumetric estimates for all levels of systolic dysfunction.

While the statistical analysis showed no significant difference in ESV, EDV, or EF values between the two methods for the population as a whole, there are several potential explanations for differences between the EF values for any individual subject. For instance, negative intrathoracic pressure of inspiration has been shown to decrease the EF inspite of increased volume due to increased transmural pressures and afterload, especially in patients with respiratory distress syndrome, asthma, and COPD [28,29]. Additionally, slice positions can change during the collection of images when using a free-breathing approach. Such motion may introduce small changes in the orientation of the heart within the chest, which could also result in differences in calculated EF values. Finally, the presence of arrhythmias in several cases and the resulting artifacts may lead to errors in the volumetric analysis. However, despite these potential sources of differences between the EF values as measured with free-breathing and breath-hold methods, only small deviations were seen between different scans in the same subject.

The statistical analysis performed based on reviewer ratings of artifacts demonstrated that the use of the radial GRAPPA approach results in fewer image artifacts (p < 0.01 based on the two-sample test of proportion that could adversely affect functional assessment of the left ventricle. The presence of fewer artifacts in the radial GRAPPA images is most likely due to the lack of gating and breath-holding in the real-time scans; no misgating or failed breath-holding can occur when no gating or breath-holding is used.

The average scan times were significantly shorter (p < 0.001) when using the real-time radial GRAPPA approach, despite the need for calibration data. The shorter scan times were due to the lack of a need for breath-holding and recovery periods. In cases where only an evaluation of EF is needed, the use of the real-time method may make CMR more rapid, accurate, and cost-effective when compared to other methods of measuring EF. While only EF evaluation was explored in this study, radial GRAPPA can be applied to accelerate other portions of the CMR examination [30,31]. If such a real-time method were employed for the entire CMR study, significant and meaningful reduction in scan time could be achieved. As other CMR researchers have shown, such a reduction in scan time has several advantages including, but not limited to, improved patient comfort and reduction in CMR and overall costs [11]. The true impact of real-time CMR may be the removal of the requirement of a steady cardiac rhythm and the ability to breath-hold, which could potentially lead to improved image quality in patients experiencing arrhythmias and who cannot hold their breath. Based on the results of this study and other similar studies using real-time CMR for patients with arrhythmia [16], it may be possible to measure EF in these patients using such real-time techniques.

The reviewer ratings of the image features indicate that the gold-standard cine imaging method offers superior visualization of several specific image features than the radial GRAPPA approach. Previous qualitative assessments of real-time CMR images reconstructed with other techniques have shown that real-time image quality is slightly [16] to moderately [12,13] worse than the standard functional images. The real-time radial GRAPPA images shown here have similar image quality for some features, and had only a modest drop in ratings for other features, when compared to the standard images. Overall, the ratings for the real-time images generated in this study were high, averaging between “good” and “excellent” for all categories. For features including myocardium, endocardial border, and cardiac motion, all related to ejection fraction, statistical tests showed that there was no significant difference between the gold-standard method and real-time imaging with radial GRAPPA. However, the analysis indicates that standard cine scans should be performed when specific anatomical structures (i.e. the mitral valve) must be assessed, when patients can provide the requisite breath-holds.

One limitation of the through-time radial GRAPPA technique is the need for a potentially lengthy calibration phase, although this data collection can occur without breath-holding or ECG gating. In the case of 26 calibration frames, the calibration requires approximately four times longer than the collection of the actual imaging data. While using only six calibration frames greatly reduces the scan time, the resulting images may be blurry due to the need for a larger k-space segment for calibration [17]. Indeed, the statistical analysis of the ratings has shown that the use of more calibration frames for the radial GRAPPA reconstruction (26 vs. 6) may lead to better image quality when looking at some specific image features, such as endocardial border definition and visualization of the papillary muscles. In cases where these features are to be assessed in addition to calculating the ejection fraction, the use of radial GRAPPA with more calibration frames may be preferred despite the longer scan time. However, for both types of real-time calibration (i.e. with 26 and 6 frames), the overall scan times were significantly shorter than the standard clinical scan, even with the collection of calibration data included. A comparison between through-time radial GRAPPA and other real-time cardiac imaging techniques which do not require large amounts of calibration data has not been performed. However, the potential advantages of through-time radial GRAPPA, including the high temporal resolution without temporal regularization or view-sharing and capability for true real-time image reconstruction, make this technique attractive despite the need for calibration data.

### Clinical implications

The results from this study build the foundation for further studies to explore real-time cardiac imaging with radial GRAPPA for the determination of EF in specific populations where current gold-standard CMR methods may be challenging, such as in patients with severe arrhythmia or with severe inability to breath-hold. It may also be useful in children who currently require general anesthesia to facilitate acquisition of sequences that require breath-holding.

## Conclusion

Real-time CMR using through-time radial GRAPPA method has been shown to yield quantitative left ventricular functional parameters equivalent to the gold-standard technique in an overall shorter scan time. While reviewers overall preferred the standard images, the radial GRAPPA images were also generally rated to have “excellent” to “good” image quality, and the radial GRAPPA images exhibited fewer artifacts as compared to gold-standard images. Based on this validation study, it may be possible to replace traditional cine techniques with the radial GRAPPA approach in cases where only EF assessment is required, and to use real-time imaging with radial GRAPPA for EF evaluation in patients with difficulties breath-holding or arrhythmias.

## Abbreviations

CMR: Cardiovascular Magnetic Resonance
GRAPPA: GeneRalized Autocalibrating Partially Parallel Acquisitions
HIPAA: The Health Insurance Portability and Accountability Act of 1996
IRB: Institutional Review Board
bSSFP: Balanced steady-state free precession
DICOM: Digital Imaging and Communications in Medicine
NUFFT: Non-Uniform Fast Fourier Transform
EDV: End-Diastolic Volume
ESV: End-Systolic Volume
EF: Ejection fraction
GPU: Graphics Processing Unit
